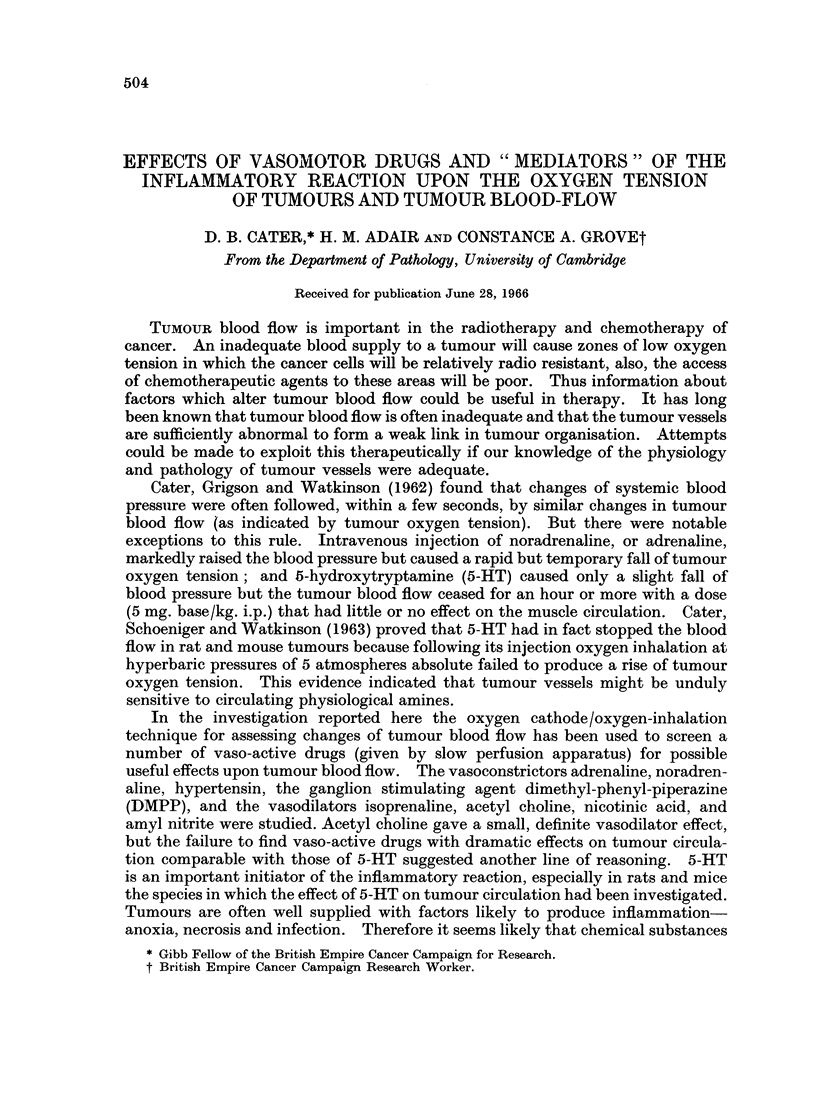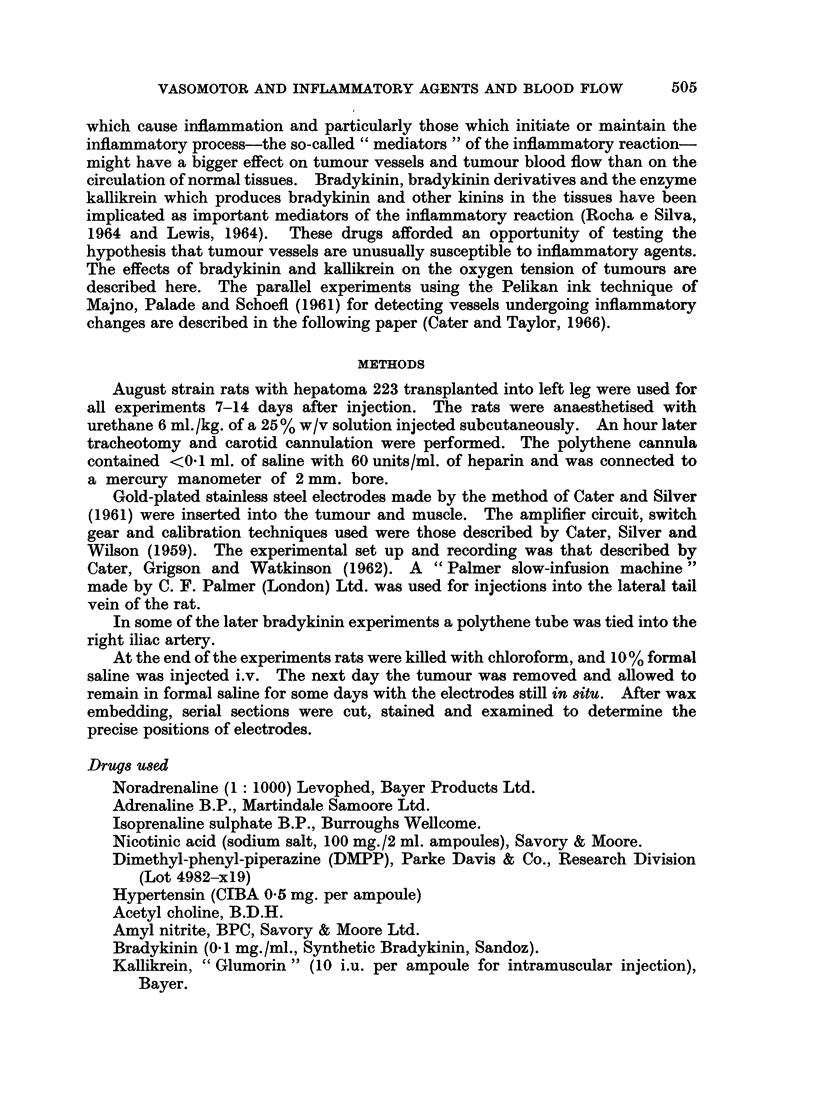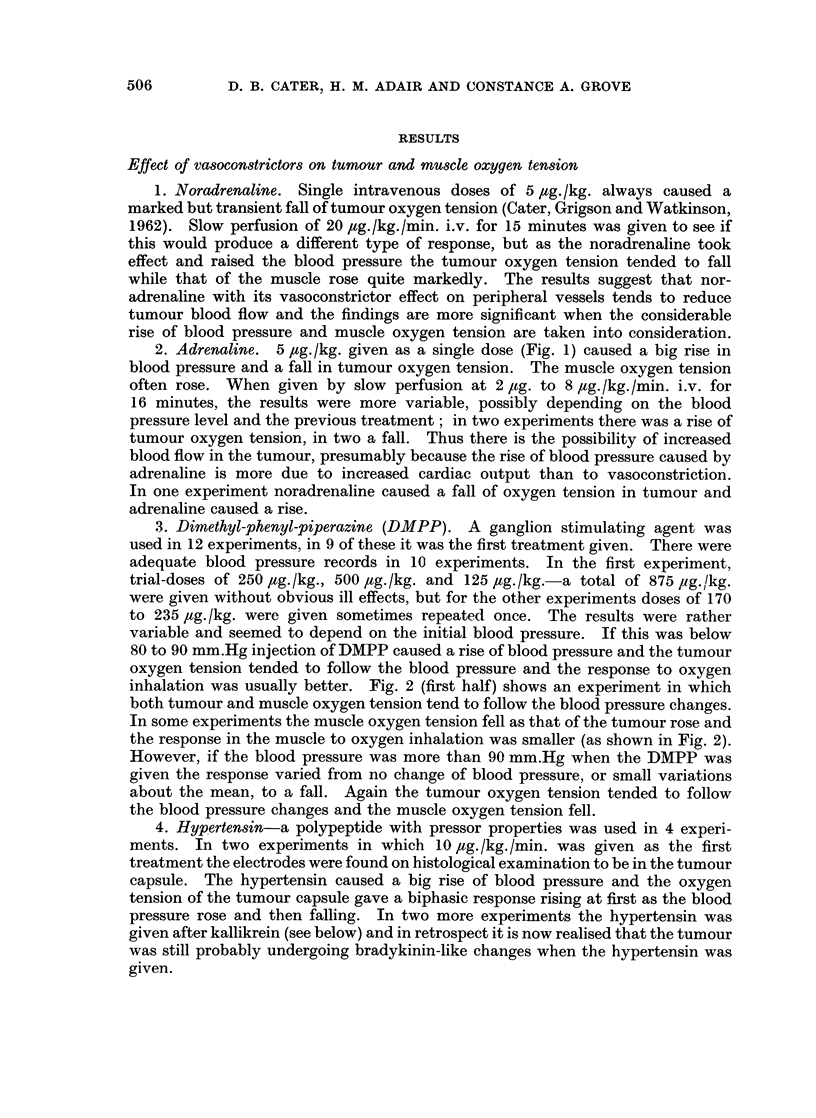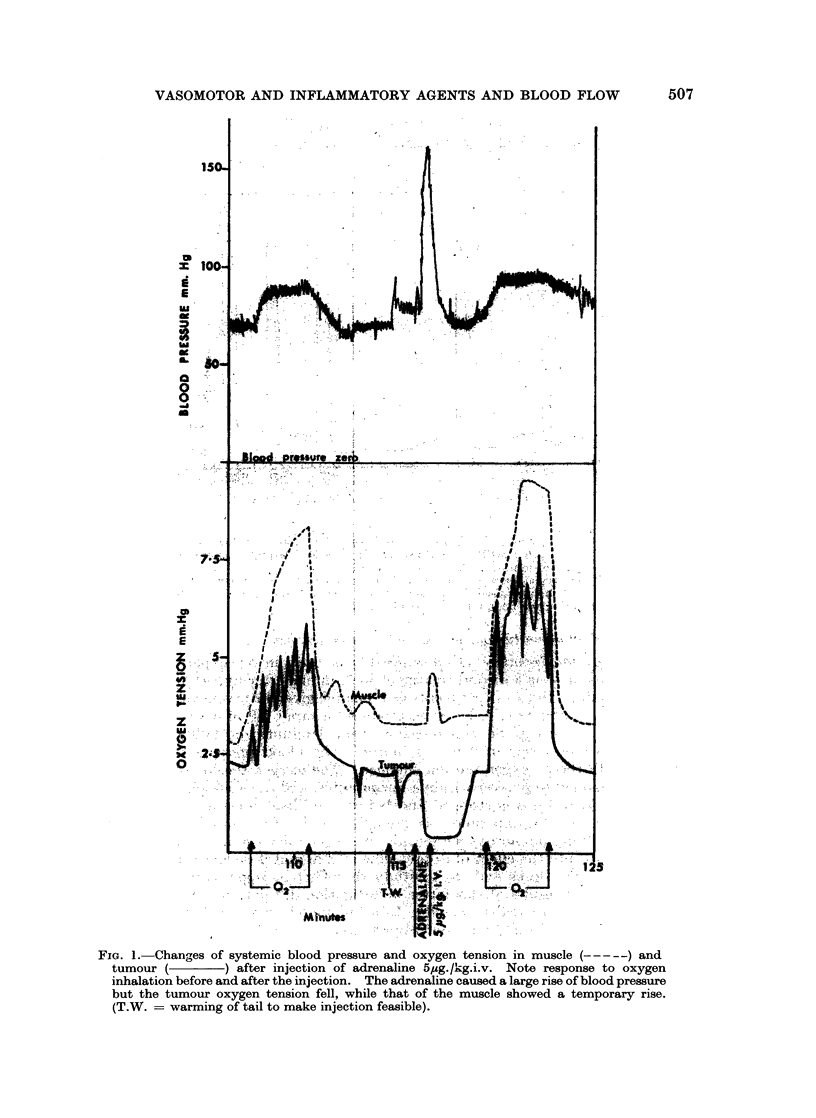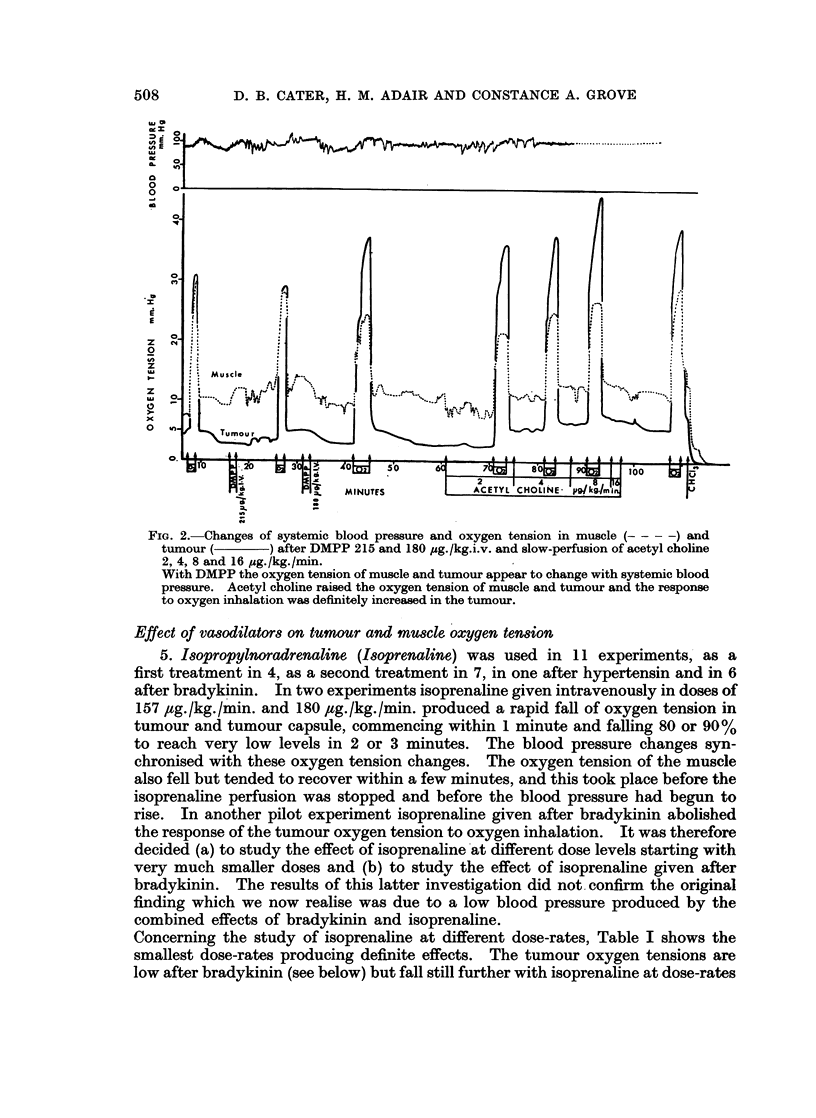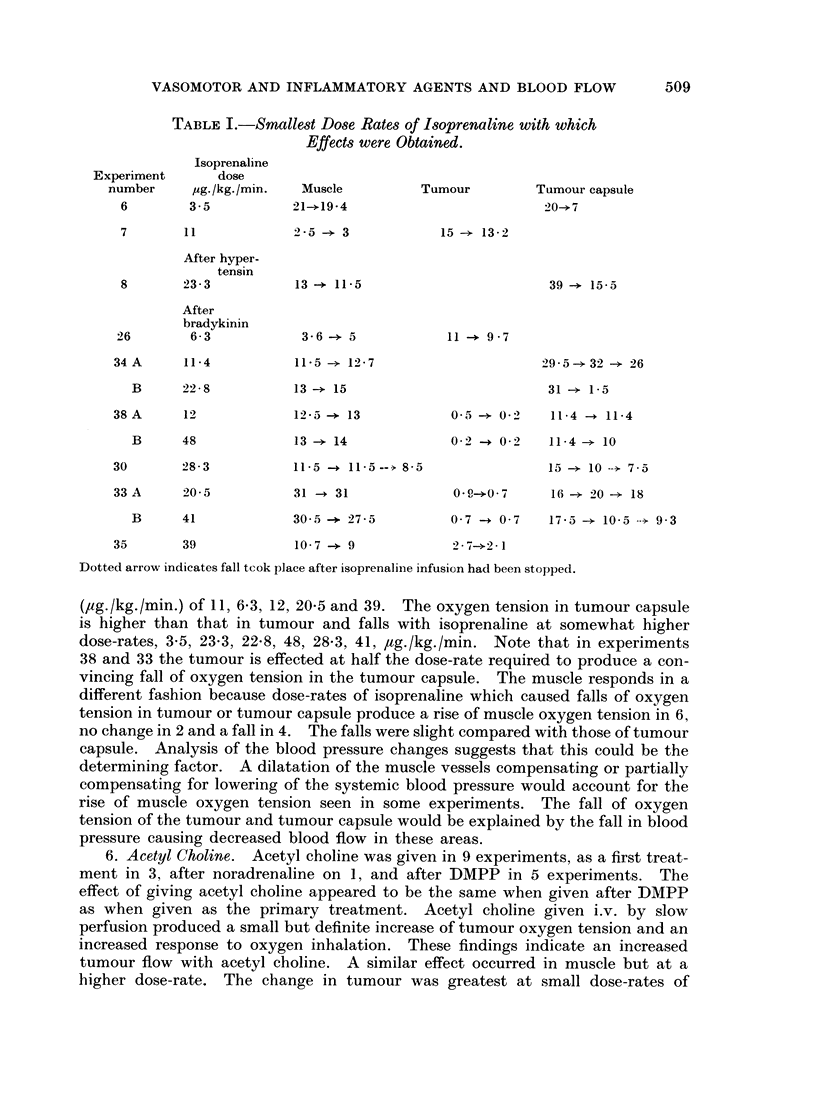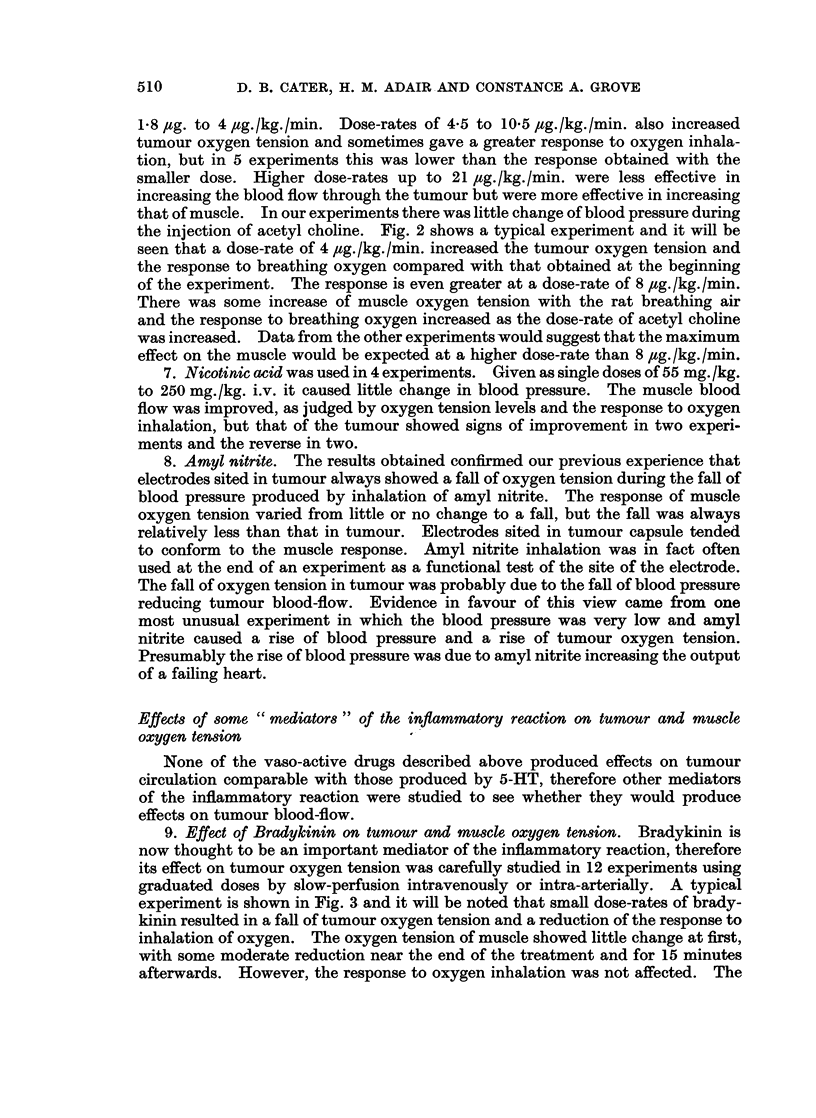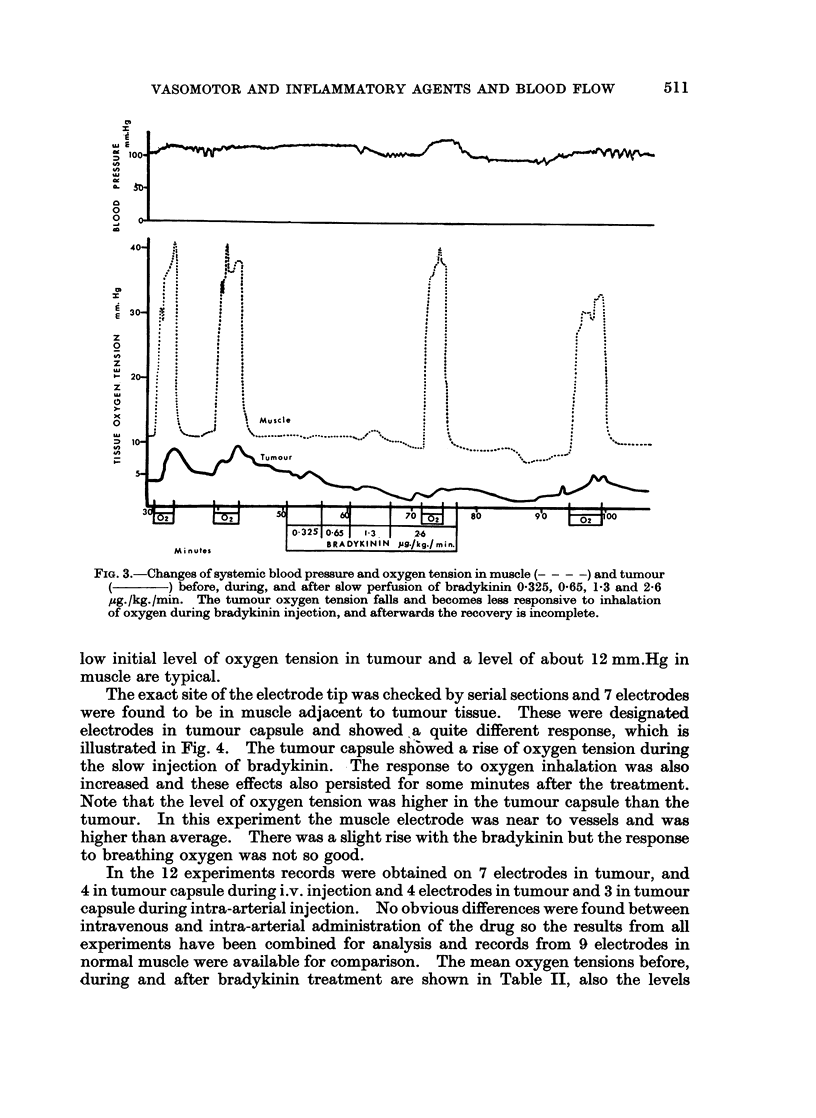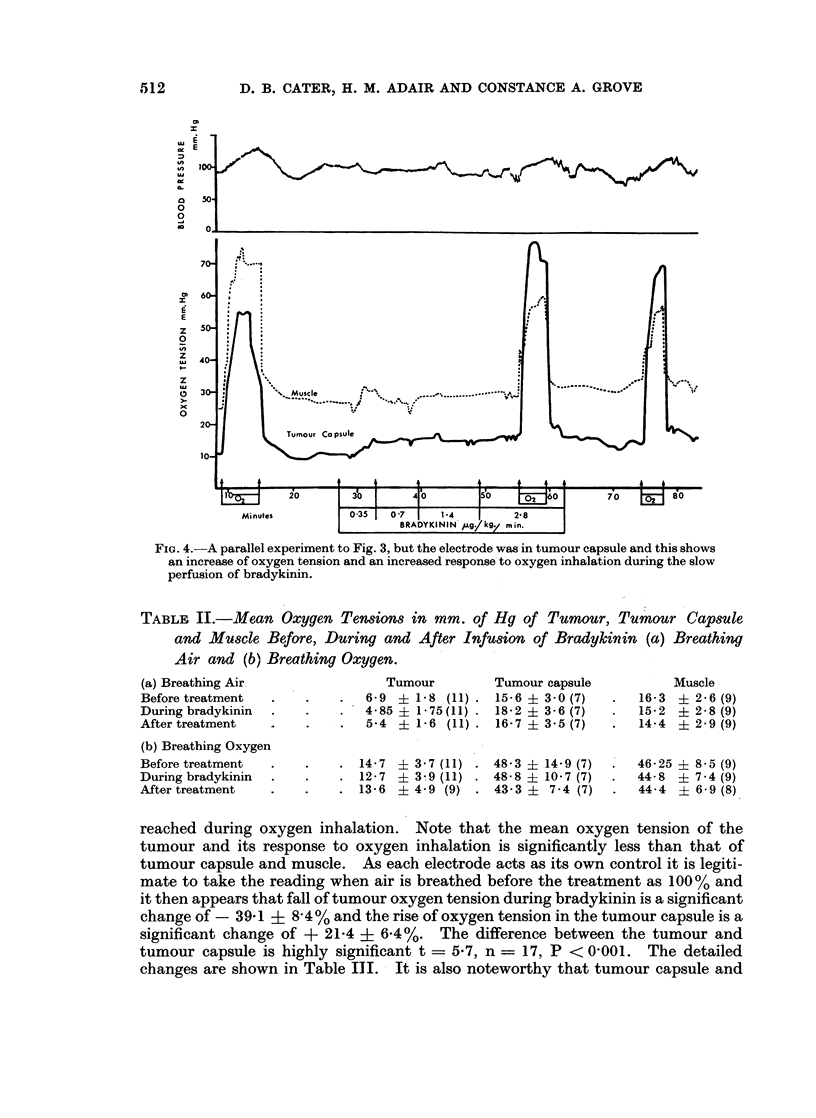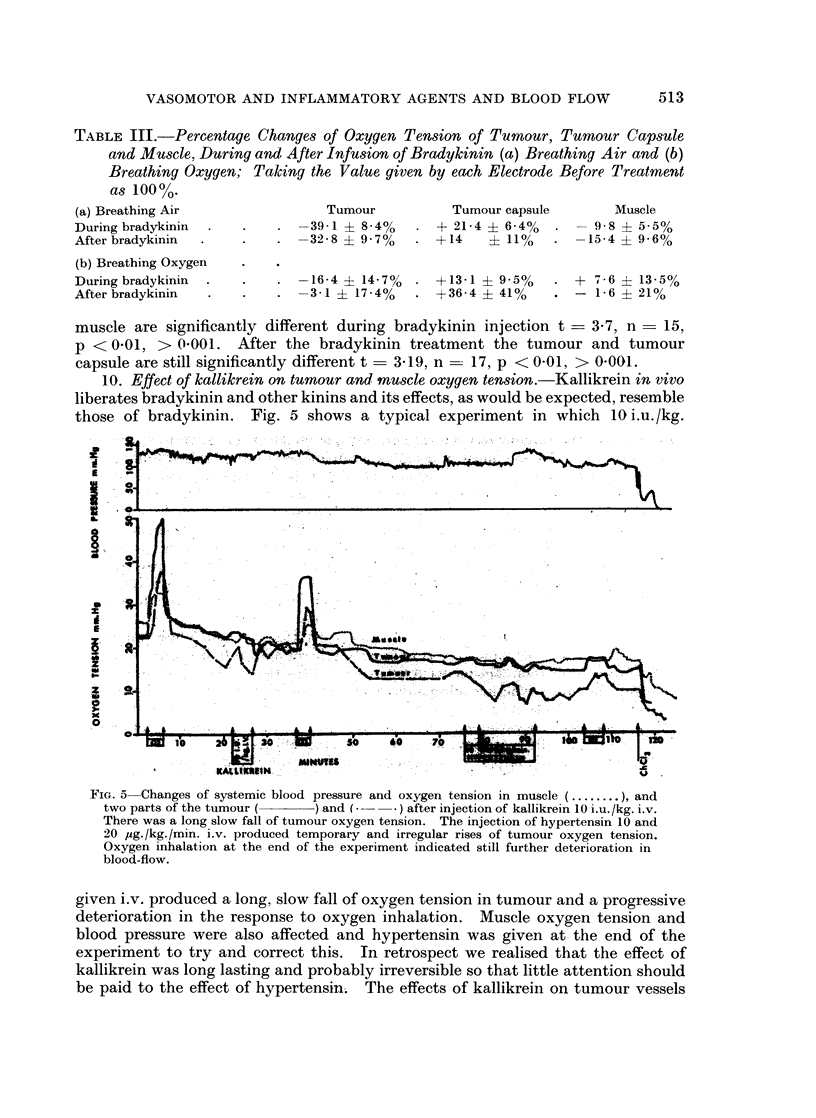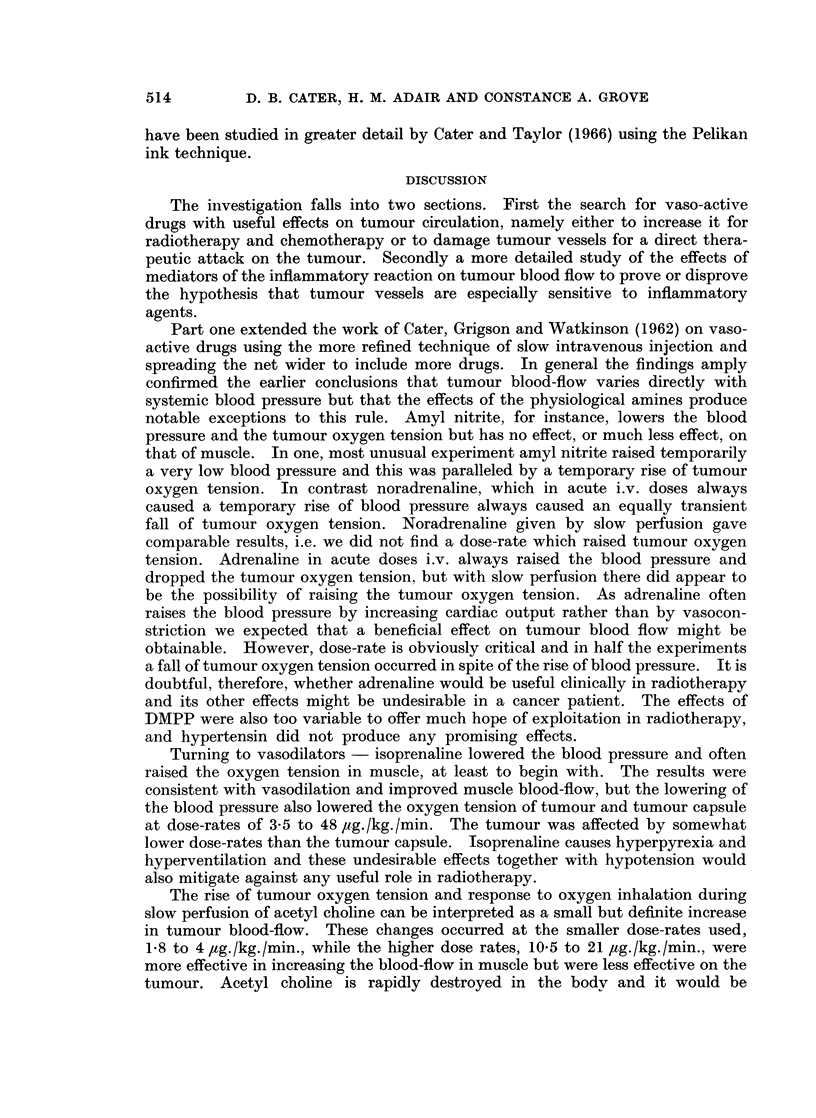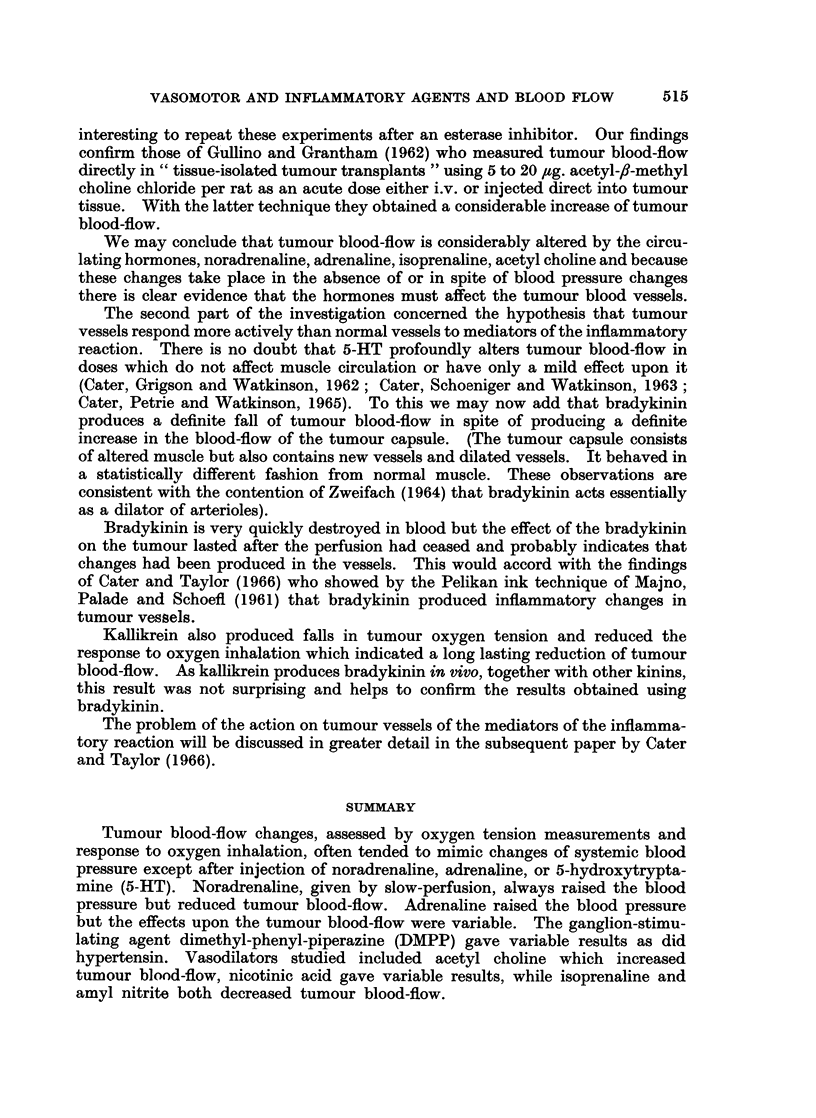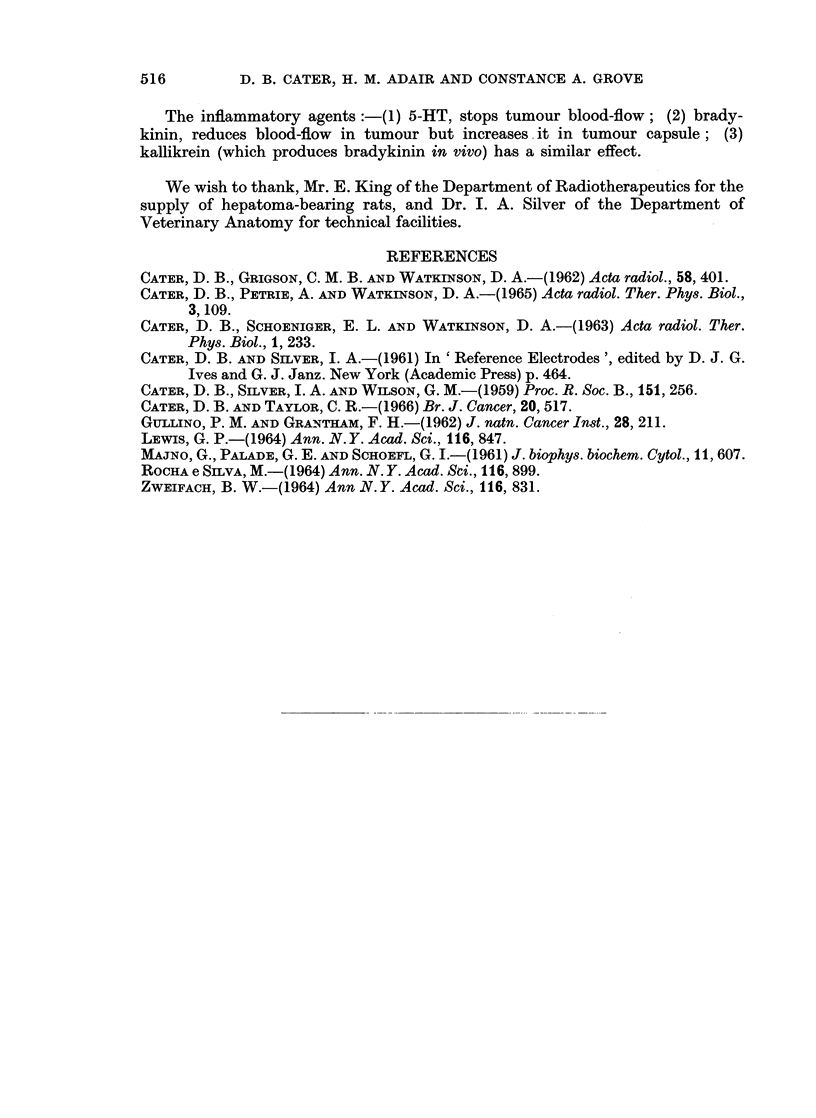# Effects of vasomotor drugs and "mediators" of the inflammatory reaction upon the oxygen tension of tumours and tumour blood-flow.

**DOI:** 10.1038/bjc.1966.62

**Published:** 1966-09

**Authors:** D. B. Cater, H. M. Adair, C. A. Grove


### EFFECTS OF VASOMOTOR DRUGS AND " MEDIATORS " OF THE INFLAMMATORY REACTION UPON THE OXYGEN TENSION OF TUMOURS AND TUMOUR BLOOD-FLOW

D. B. CATER,* H. M. ADAIR AND CONSTANCE A. GROVE†

*From the Department of Pathology, University of Cambridge*

Received for publication June 28, 1966

TUMOUR blood flow is important in the radiotherapy and chemotherapy of cancer. An inadequate blood supply to a tumour will cause zones of low oxygen tension in which the cancer cells will be relatively radio resistant, also, the access of chemotherapeutic agents to these areas will be poor. Thus information about factors which alter tumour blood flow could be useful in therapy. It has long been known that tumour blood flow is often inadequate and that the tumour vessels are sufficiently abnormal to form a weak link in tumour organisation. Attempts could be made to exploit this therapeutically if our knowledge of the physiology and pathology of tumour vessels were adequate.

Cater, Grigson and Watkinson (1962) found that changes of systemic blood pressure were often followed, within a few seconds, by similar changes in tumour blood flow (as indicated by tumour oxygen tension). But there were notable exceptions to this rule. Intravenous injection of noradrenaline, or adrenaline, markedly raised the blood pressure but caused a rapid but temporary fall of tumour oxygen tension ; and 5-hydroxytryptamine (5-HT) caused only a slight fall of blood pressure but the tumour blood flow ceased for an hour or more with a dose (5 mg. base/kg. i.p.) that had little or no effect on the muscle circulation. Cater, Schoeniger and Watkinson (1963) proved that 5-HT had in fact stopped the blood flow in rat and mouse tumours because following its injection oxygen inhalation at hyperbaric pressures of 5 atmospheres absolute failed to produce a rise of tumour oxygen tension. This evidence indicated that tumour vessels might be unduly sensitive to circulating physiological amines.

In the investigation reported here the oxygen cathode/oxygen-inhalation technique for assessing changes of tumour blood flow has been used to screen a number of vaso-active drugs (given by slow perfusion apparatus) for possible useful effects upon tumour blood flow. The vasoconstrictors adrenaline, noradrenaline, hypertensin, the ganglion stimulating agent dimethyl-phenyl-piperazine (DMPP), and the vasodilators isoprenaline, acetyl choline, nicotinic acid, and amyl nitrite were studied. Acetyl choline gave a small, definite vasodilator effect, but the failure to find vaso-active drugs with dramatic effects on tumour circulation comparable with those of 5-HT suggested another line of reasoning. 5-HT is an important initiator of the inflammatory reaction, especially in rats and mice the species in which the effect of 5-HT on tumour circulation had been investigated. Tumours are often well supplied with factors likely to produce inflammation—anoxia, necrosis and infection. Therefore it seems likely that chemical substances

* Gibb Fellow of the British Empire Cancer Campaign for Research.
† British Empire Cancer Campaign Research Worker.

which cause inflammation and particularly those which initiate or maintain the inflammatory process—the so-called " mediators " of the inflammatory reaction—might have a bigger effect on tumour vessels and tumour blood flow than on the circulation of normal tissues. Bradykinin, bradykinin derivatives and the enzyme kallikrein which produces bradykinin and other kinins in the tissues have been implicated as important mediators of the inflammatory reaction (Rocha e Silva, 1964 and Lewis, 1964). These drugs afforded an opportunity of testing the hypothesis that tumour vessels are unusually susceptible to inflammatory agents. The effects of bradykinin and kallikrein on the oxygen tension of tumours are described here. The parallel experiments using the Pelikan ink technique of Majno, Palade and Schoefl (1961) for detecting vessels undergoing inflammatory changes are described in the following paper (Cater and Taylor, 1966).

### METHODS

August strain rats with hepatoma 223 transplanted into left leg were used for all experiments 7–14 days after injection. The rats were anaesthetised with urethane 6 ml./kg. of a 25% w/v solution injected subcutaneously. An hour later tracheotomy and carotid cannulation were performed. The polythene cannula contained <0·1 ml. of saline with 60 units/ml. of heparin and was connected to a mercury manometer of 2 mm. bore.

Gold-plated stainless steel electrodes made by the method of Cater and Silver (1961) were inserted into the tumour and muscle. The amplifier circuit, switch gear and calibration techniques used were those described by Cater, Silver and Wilson (1959). The experimental set up and recording was that described by Cater, Grigson and Watkinson (1962). A " Palmer slow-infusion machine " made by C. F. Palmer (London) Ltd. was used for injections into the lateral tail vein of the rat.

In some of the later bradykinin experiments a polythene tube was tied into the right iliac artery.

At the end of the experiments rats were killed with chloroform, and 10% formal saline was injected i.v. The next day the tumour was removed and allowed to remain in formal saline for some days with the electrodes still *in situ*. After wax embedding, serial sections were cut, stained and examined to determine the precise positions of electrodes.

### *Drugs used*

Noradrenaline (1 : 1000) Levophed, Bayer Products Ltd.
Adrenaline B.P., Martindale Samoore Ltd.
Isoprenaline sulphate B.P., Burroughs Wellcome.
Nicotinic acid (sodium salt, 100 mg./2 ml. ampoules), Savory & Moore.
Dimethyl-phenyl-piperazine (DMPP), Parke Davis & Co., Research Division (Lot 4982–x19)
Hypertensin (CIBA 0·5 mg. per ampoule)
Acetyl choline, B.D.H.
Amyl nitrite, BPC, Savory & Moore Ltd.
Bradykinin (0·1 mg./ml., Synthetic Bradykinin, Sandoz).
Kallikrein, " Glumorin " (10 i.u. per ampoule for intramuscular injection), Bayer.

### RESULTS

### *Effect of vasoconstrictors on tumour and muscle oxygen tension*

1. *Noradrenaline.* Single intravenous doses of 5 μg./kg. always caused a marked but transient fall of tumour oxygen tension (Cater, Grigson and Watkinson, 1962). Slow perfusion of 20 μg./kg./min. i.v. for 15 minutes was given to see if this would produce a different type of response, but as the noradrenaline took effect and raised the blood pressure the tumour oxygen tension tended to fall while that of the muscle rose quite markedly. The results suggest that noradrenaline with its vasoconstrictor effect on peripheral vessels tends to reduce tumour blood flow and the findings are more significant when the considerable rise of blood pressure and muscle oxygen tension are taken into consideration.

2. *Adrenaline.* 5 μg./kg. given as a single dose (Fig. 1) caused a big rise in blood pressure and a fall in tumour oxygen tension. The muscle oxygen tension often rose. When given by slow perfusion at 2 μg. to 8 μg./kg./min. i.v. for 16 minutes, the results were more variable, possibly depending on the blood pressure level and the previous treatment ; in two experiments there was a rise of tumour oxygen tension, in two a fall. Thus there is the possibility of increased blood flow in the tumour, presumably because the rise of blood pressure caused by adrenaline is more due to increased cardiac output than to vasoconstriction. In one experiment noradrenaline caused a fall of oxygen tension in tumour and adrenaline caused a rise.

3. *Dimethyl-phenyl-piperazine (DMPP).* A ganglion stimulating agent was used in 12 experiments, in 9 of these it was the first treatment given. There were adequate blood pressure records in 10 experiments. In the first experiment, trial-doses of 250 μg./kg., 500 μg./kg. and 125 μg./kg.—a total of 875 μg./kg. were given without obvious ill effects, but for the other experiments doses of 170 to 235 μg./kg. were given sometimes repeated once. The results were rather variable and seemed to depend on the initial blood pressure. If this was below 80 to 90 mm.Hg injection of DMPP caused a rise of blood pressure and the tumour oxygen tension tended to follow the blood pressure and the response to oxygen inhalation was usually better. Fig. 2 (first half) shows an experiment in which both tumour and muscle oxygen tension tend to follow the blood pressure changes. In some experiments the muscle oxygen tension fell as that of the tumour rose and the response in the muscle to oxygen inhalation was smaller (as shown in Fig. 2). However, if the blood pressure was more than 90 mm.Hg when the DMPP was given the response varied from no change of blood pressure, or small variations about the mean, to a fall. Again the tumour oxygen tension tended to follow the blood pressure changes and the muscle oxygen tension fell.

4. *Hypertensin*—a polypeptide with pressor properties was used in 4 experiments. In two experiments in which 10 μg./kg./min. was given as the first treatment the electrodes were found on histological examination to be in the tumour capsule. The hypertensin caused a big rise of blood pressure and the oxygen tension of the tumour capsule gave a biphasic response rising at first as the blood pressure rose and then falling. In two more experiments the hypertensin was given after kallikrein (see below) and in retrospect it is now realised that the tumour was still probably undergoing bradykinin-like changes when the hypertensin was given.

FIG. 1.—Changes of systemic blood pressure and oxygen tension in muscle (-----) and tumour (————) after injection of adrenaline 5$\mu$g./kg.i.v. Note response to oxygen inhalation before and after the injection. The adrenaline caused a large rise of blood pressure but the tumour oxygen tension fell, while that of the muscle showed a temporary rise. (T.W. = warming of tail to make injection feasible).

FIG. 2.—Changes of systemic blood pressure and oxygen tension in muscle (– – – –) and tumour (————) after DMPP 215 and 180 µg./kg.i.v. and slow-perfusion of acetyl choline 2, 4, 8 and 16 µg./kg./min.

With DMPP the oxygen tension of muscle and tumour appear to change with systemic blood pressure. Acetyl choline raised the oxygen tension of muscle and tumour and the response to oxygen inhalation was definitely increased in the tumour.

*Effect of vasodilators on tumour and muscle oxygen tension*

5. *Isopropylnoradrenaline* (*Isoprenaline*) was used in 11 experiments, as a first treatment in 4, as a second treatment in 7, in one after hypertensin and in 6 after bradykinin. In two experiments isoprenaline given intravenously in doses of 157 µg./kg./min. and 180 µg./kg./min. produced a rapid fall of oxygen tension in tumour and tumour capsule, commencing within 1 minute and falling 80 or 90% to reach very low levels in 2 or 3 minutes. The blood pressure changes synchronised with these oxygen tension changes. The oxygen tension of the muscle also fell but tended to recover within a few minutes, and this took place before the isoprenaline perfusion was stopped and before the blood pressure had begun to rise. In another pilot experiment isoprenaline given after bradykinin abolished the response of the tumour oxygen tension to oxygen inhalation. It was therefore decided (a) to study the effect of isoprenaline at different dose levels starting with very much smaller doses and (b) to study the effect of isoprenaline given after bradykinin. The results of this latter investigation did not confirm the original finding which we now realise was due to a low blood pressure produced by the combined effects of bradykinin and isoprenaline.

Concerning the study of isoprenaline at different dose-rates, Table I shows the smallest dose-rates producing definite effects. The tumour oxygen tensions are low after bradykinin (see below) but fall still further with isoprenaline at dose-rates

TABLE I.—*Smallest Dose Rates of Isoprenaline with which Effects were Obtained.*

| Experiment number | Isoprenaline dose µg./kg./min. | Muscle | Tumour | Tumour capsule |
|---|---|---|---|---|
| 6 | 3·5 | 21→19·4 | | 20→7 |
| 7 | 11 | 2·5 → 3 | 15 → 13·2 | |
| 8 | After hyper-tensin 23·3 | 13 → 11·5 | | 39 → 15·5 |
| 26 | After bradykinin 6·3 | 3·6 → 5 | 11 → 9·7 | |
| 34 A | 11·4 | 11·5 → 12·7 | | 29·5 → 32 → 26 |
| B | 22·8 | 13 → 15 | | 31 → 1·5 |
| 38 A | 12 | 12·5 → 13 | 0·5 → 0·2 | 11·4 → 11·4 |
| B | 48 | 13 → 14 | 0·2 → 0·2 | 11·4 → 10 |
| 30 | 28·3 | 11·5 → 11·5 --> 8·5 | | 15 → 10 --> 7·5 |
| 33 A | 20·5 | 31 → 31 | 0·9→0·7 | 16 → 20 → 18 |
| B | 41 | 30·5 → 27·5 | 0·7 → 0·7 | 17·5 → 10·5 --> 9·3 |
| 35 | 39 | 10·7 → 9 | 2·7→2·1 | |

Dotted arrow indicates fall took place after isoprenaline infusion had been stopped.

(µg./kg./min.) of 11, 6·3, 12, 20·5 and 39. The oxygen tension in tumour capsule is higher than that in tumour and falls with isoprenaline at somewhat higher dose-rates, 3·5, 23·3, 22·8, 48, 28·3, 41, µg./kg./min. Note that in experiments 38 and 33 the tumour is effected at half the dose-rate required to produce a convincing fall of oxygen tension in the tumour capsule. The muscle responds in a different fashion because dose-rates of isoprenaline which caused falls of oxygen tension in tumour or tumour capsule produce a rise of muscle oxygen tension in 6, no change in 2 and a fall in 4. The falls were slight compared with those of tumour capsule. Analysis of the blood pressure changes suggests that this could be the determining factor. A dilatation of the muscle vessels compensating or partially compensating for lowering of the systemic blood pressure would account for the rise of muscle oxygen tension seen in some experiments. The fall of oxygen tension of the tumour and tumour capsule would be explained by the fall in blood pressure causing decreased blood flow in these areas.

6. *Acetyl Choline.* Acetyl choline was given in 9 experiments, as a first treatment in 3, after noradrenaline on 1, and after DMPP in 5 experiments. The effect of giving acetyl choline appeared to be the same when given after DMPP as when given as the primary treatment. Acetyl choline given i.v. by slow perfusion produced a small but definite increase of tumour oxygen tension and an increased response to oxygen inhalation. These findings indicate an increased tumour flow with acetyl choline. A similar effect occurred in muscle but at a higher dose-rate. The change in tumour was greatest at small dose-rates of

1·8 $\mu$g. to 4 $\mu$g./kg./min. Dose-rates of 4·5 to 10·5 $\mu$g./kg./min. also increased tumour oxygen tension and sometimes gave a greater response to oxygen inhalation, but in 5 experiments this was lower than the response obtained with the smaller dose. Higher dose-rates up to 21 $\mu$g./kg./min. were less effective in increasing the blood flow through the tumour but were more effective in increasing that of muscle. In our experiments there was little change of blood pressure during the injection of acetyl choline. Fig. 2 shows a typical experiment and it will be seen that a dose-rate of 4 $\mu$g./kg./min. increased the tumour oxygen tension and the response to breathing oxygen compared with that obtained at the beginning of the experiment. The response is even greater at a dose-rate of 8 $\mu$g./kg./min. There was some increase of muscle oxygen tension with the rat breathing air and the response to breathing oxygen increased as the dose-rate of acetyl choline was increased. Data from the other experiments would suggest that the maximum effect on the muscle would be expected at a higher dose-rate than 8 $\mu$g./kg./min.

7. *Nicotinic acid* was used in 4 experiments. Given as single doses of 55 mg./kg. to 250 mg./kg. i.v. it caused little change in blood pressure. The muscle blood flow was improved, as judged by oxygen tension levels and the response to oxygen inhalation, but that of the tumour showed signs of improvement in two experiments and the reverse in two.

8. *Amyl nitrite.* The results obtained confirmed our previous experience that electrodes sited in tumour always showed a fall of oxygen tension during the fall of blood pressure produced by inhalation of amyl nitrite. The response of muscle oxygen tension varied from little or no change to a fall, but the fall was always relatively less than that in tumour. Electrodes sited in tumour capsule tended to conform to the muscle response. Amyl nitrite inhalation was in fact often used at the end of an experiment as a functional test of the site of the electrode. The fall of oxygen tension in tumour was probably due to the fall of blood pressure reducing tumour blood-flow. Evidence in favour of this view came from one most unusual experiment in which the blood pressure was very low and amyl nitrite caused a rise of blood pressure and a rise of tumour oxygen tension. Presumably the rise of blood pressure was due to amyl nitrite increasing the output of a failing heart.

*Effects of some "mediators" of the inflammatory reaction on tumour and muscle oxygen tension*

None of the vaso-active drugs described above produced effects on tumour circulation comparable with those produced by 5-HT, therefore other mediators of the inflammatory reaction were studied to see whether they would produce effects on tumour blood-flow.

9. *Effect of Bradykinin on tumour and muscle oxygen tension.* Bradykinin is now thought to be an important mediator of the inflammatory reaction, therefore its effect on tumour oxygen tension was carefully studied in 12 experiments using graduated doses by slow-perfusion intravenously or intra-arterially. A typical experiment is shown in Fig. 3 and it will be noted that small dose-rates of bradykinin resulted in a fall of tumour oxygen tension and a reduction of the response to inhalation of oxygen. The oxygen tension of muscle showed little change at first, with some moderate reduction near the end of the treatment and for 15 minutes afterwards. However, the response to oxygen inhalation was not affected. The

FIG. 3.—Changes of systemic blood pressure and oxygen tension in muscle (– – – –) and tumour (————) before, during, and after slow perfusion of bradykinin 0·325, 0·65, 1·3 and 2·6 μg./kg./min. The tumour oxygen tension falls and becomes less responsive to inhalation of oxygen during bradykinin injection, and afterwards the recovery is incomplete.

low initial level of oxygen tension in tumour and a level of about 12 mm.Hg in muscle are typical.

The exact site of the electrode tip was checked by serial sections and 7 electrodes were found to be in muscle adjacent to tumour tissue. These were designated electrodes in tumour capsule and showed a quite different response, which is illustrated in Fig. 4. The tumour capsule showed a rise of oxygen tension during the slow injection of bradykinin. The response to oxygen inhalation was also increased and these effects also persisted for some minutes after the treatment. Note that the level of oxygen tension was higher in the tumour capsule than the tumour. In this experiment the muscle electrode was near to vessels and was higher than average. There was a slight rise with the bradykinin but the response to breathing oxygen was not so good.

In the 12 experiments records were obtained on 7 electrodes in tumour, and 4 in tumour capsule during i.v. injection and 4 electrodes in tumour and 3 in tumour capsule during intra-arterial injection. No obvious differences were found between intravenous and intra-arterial administration of the drug so the results from all experiments have been combined for analysis and records from 9 electrodes in normal muscle were available for comparison. The mean oxygen tensions before, during and after bradykinin treatment are shown in Table II, also the levels

FIG. 4.—A parallel experiment to Fig. 3, but the electrode was in tumour capsule and this shows an increase of oxygen tension and an increased response to oxygen inhalation during the slow perfusion of bradykinin.

TABLE II.—*Mean Oxygen Tensions in mm. of Hg of Tumour, Tumour Capsule and Muscle Before, During and After Infusion of Bradykinin (a) Breathing Air and (b) Breathing Oxygen.*

| (a) Breathing Air | Tumour | Tumour capsule | Muscle |
|---|---|---|---|
| Before treatment | 6·9 ± 1·8 (11) | 15·6 ± 3·0 (7) | 16·3 ± 2·6 (9) |
| During bradykinin | 4·85 ± 1·75 (11) | 18·2 ± 3·6 (7) | 15·2 ± 2·8 (9) |
| After treatment | 5·4 ± 1·6 (11) | 16·7 ± 3·5 (7) | 14·4 ± 2·9 (9) |
| (b) Breathing Oxygen | | | |
| Before treatment | 14·7 ± 3·7 (11) | 48·3 ± 14·9 (7) | 46·25 ± 8·5 (9) |
| During bradykinin | 12·7 ± 3·9 (11) | 48·8 ± 10·7 (7) | 44·8 ± 7·4 (9) |
| After treatment | 13·6 ± 4·9 (9) | 43·3 ± 7·4 (7) | 44·4 ± 6·9 (8) |

reached during oxygen inhalation. Note that the mean oxygen tension of the tumour and its response to oxygen inhalation is significantly less than that of tumour capsule and muscle. As each electrode acts as its own control it is legitimate to take the reading when air is breathed before the treatment as 100% and it then appears that fall of tumour oxygen tension during bradykinin is a significant change of — 39·1 ± 8·4% and the rise of oxygen tension in the tumour capsule is a significant change of + 21·4 ± 6·4%. The difference between the tumour and tumour capsule is highly significant $t = 5{\cdot}7$, $n = 17$, $P < 0{\cdot}001$. The detailed changes are shown in Table III. It is also noteworthy that tumour capsule and

TABLE III.—*Percentage Changes of Oxygen Tension of Tumour, Tumour Capsule and Muscle, During and After Infusion of Bradykinin (a) Breathing Air and (b) Breathing Oxygen; Taking the Value given by each Electrode Before Treatment as* 100%.

| (a) Breathing Air | Tumour | Tumour capsule | Muscle |
|---|---|---|---|
| During bradykinin | −39·1 ± 8·4% | + 21·4 ± 6·4% | − 9·8 ± 5·5% |
| After bradykinin | −32·8 ± 9·7% | +14 ± 11% | −15·4 ± 9·6% |
| (b) Breathing Oxygen | | | |
| During bradykinin | −16·4 ± 14·7% | +13·1 ± 9·5% | + 7·6 ± 13·5% |
| After bradykinin | −3·1 ± 17·4% | +36·4 ± 41% | − 1·6 ± 21% |

muscle are significantly different during bradykinin injection $t = 3{\cdot}7$, $n = 15$, $p < 0{\cdot}01, > 0{\cdot}001$. After the bradykinin treatment the tumour and tumour capsule are still significantly different $t = 3{\cdot}19$, $n = 17$, $p < 0{\cdot}01, > 0{\cdot}001$.

10. *Effect of kallikrein on tumour and muscle oxygen tension.*—Kallikrein *in vivo* liberates bradykinin and other kinins and its effects, as would be expected, resemble those of bradykinin. Fig. 5 shows a typical experiment in which 10 i.u./kg.

FIG. 5—Changes of systemic blood pressure and oxygen tension in muscle (........), and two parts of the tumour (————) and (·——·) after injection of kallikrein 10 i.u./kg. i.v. There was a long slow fall of tumour oxygen tension. The injection of hypertensin 10 and 20 µg./kg./min. i.v. produced temporary and irregular rises of tumour oxygen tension. Oxygen inhalation at the end of the experiment indicated still further deterioration in blood-flow.

given i.v. produced a long, slow fall of oxygen tension in tumour and a progressive deterioration in the response to oxygen inhalation. Muscle oxygen tension and blood pressure were also affected and hypertensin was given at the end of the experiment to try and correct this. In retrospect we realised that the effect of kallikrein was long lasting and probably irreversible so that little attention should be paid to the effect of hypertensin. The effects of kallikrein on tumour vessels have been studied in greater detail by Cater and Taylor (1966) using the Pelikan ink technique.

### DISCUSSION

The investigation falls into two sections. First the search for vaso-active drugs with useful effects on tumour circulation, namely either to increase it for radiotherapy and chemotherapy or to damage tumour vessels for a direct therapeutic attack on the tumour. Secondly a more detailed study of the effects of mediators of the inflammatory reaction on tumour blood flow to prove or disprove the hypothesis that tumour vessels are especially sensitive to inflammatory agents.

Part one extended the work of Cater, Grigson and Watkinson (1962) on vaso-active drugs using the more refined technique of slow intravenous injection and spreading the net wider to include more drugs. In general the findings amply confirmed the earlier conclusions that tumour blood-flow varies directly with systemic blood pressure but that the effects of the physiological amines produce notable exceptions to this rule. Amyl nitrite, for instance, lowers the blood pressure and the tumour oxygen tension but has no effect, or much less effect, on that of muscle. In one, most unusual experiment amyl nitrite raised temporarily a very low blood pressure and this was paralleled by a temporary rise of tumour oxygen tension. In contrast noradrenaline, which in acute i.v. doses always caused a temporary rise of blood pressure always caused an equally transient fall of tumour oxygen tension. Noradrenaline given by slow perfusion gave comparable results, i.e. we did not find a dose-rate which raised tumour oxygen tension. Adrenaline in acute doses i.v. always raised the blood pressure and dropped the tumour oxygen tension, but with slow perfusion there did appear to be the possibility of raising the tumour oxygen tension. As adrenaline often raises the blood pressure by increasing cardiac output rather than by vasoconstriction we expected that a beneficial effect on tumour blood flow might be obtainable. However, dose-rate is obviously critical and in half the experiments a fall of tumour oxygen tension occurred in spite of the rise of blood pressure. It is doubtful, therefore, whether adrenaline would be useful clinically in radiotherapy and its other effects might be undesirable in a cancer patient. The effects of DMPP were also too variable to offer much hope of exploitation in radiotherapy, and hypertensin did not produce any promising effects.

Turning to vasodilators — isoprenaline lowered the blood pressure and often raised the oxygen tension in muscle, at least to begin with. The results were consistent with vasodilation and improved muscle blood-flow, but the lowering of the blood pressure also lowered the oxygen tension of tumour and tumour capsule at dose-rates of 3·5 to 48 $\mu$g./kg./min. The tumour was affected by somewhat lower dose-rates than the tumour capsule. Isoprenaline causes hyperpyrexia and hyperventilation and these undesirable effects together with hypotension would also mitigate against any useful role in radiotherapy.

The rise of tumour oxygen tension and response to oxygen inhalation during slow perfusion of acetyl choline can be interpreted as a small but definite increase in tumour blood-flow. These changes occurred at the smaller dose-rates used, 1·8 to 4 $\mu$g./kg./min., while the higher dose rates, 10·5 to 21 $\mu$g./kg./min., were more effective in increasing the blood-flow in muscle but were less effective on the tumour. Acetyl choline is rapidly destroyed in the body and it would be interesting to repeat these experiments after an esterase inhibitor. Our findings confirm those of Gullino and Grantham (1962) who measured tumour blood-flow directly in " tissue-isolated tumour transplants " using 5 to 20 $\mu$g. acetyl-$\beta$-methyl choline chloride per rat as an acute dose either i.v. or injected direct into tumour tissue. With the latter technique they obtained a considerable increase of tumour blood-flow.

We may conclude that tumour blood-flow is considerably altered by the circulating hormones, noradrenaline, adrenaline, isoprenaline, acetyl choline and because these changes take place in the absence of or in spite of blood pressure changes there is clear evidence that the hormones must affect the tumour blood vessels.

The second part of the investigation concerned the hypothesis that tumour vessels respond more actively than normal vessels to mediators of the inflammatory reaction. There is no doubt that 5-HT profoundly alters tumour blood-flow in doses which do not affect muscle circulation or have only a mild effect upon it (Cater, Grigson and Watkinson, 1962 ; Cater, Schoeniger and Watkinson, 1963 ; Cater, Petrie and Watkinson, 1965). To this we may now add that bradykinin produces a definite fall of tumour blood-flow in spite of producing a definite increase in the blood-flow of the tumour capsule. (The tumour capsule consists of altered muscle but also contains new vessels and dilated vessels. It behaved in a statistically different fashion from normal muscle. These observations are consistent with the contention of Zweifach (1964) that bradykinin acts essentially as a dilator of arterioles).

Bradykinin is very quickly destroyed in blood but the effect of the bradykinin on the tumour lasted after the perfusion had ceased and probably indicates that changes had been produced in the vessels. This would accord with the findings of Cater and Taylor (1966) who showed by the Pelikan ink technique of Majno, Palade and Schoefl (1961) that bradykinin produced inflammatory changes in tumour vessels.

Kallikrein also produced falls in tumour oxygen tension and reduced the response to oxygen inhalation which indicated a long lasting reduction of tumour blood-flow. As kallikrein produces bradykinin *in vivo*, together with other kinins, this result was not surprising and helps to confirm the results obtained using bradykinin.

The problem of the action on tumour vessels of the mediators of the inflammatory reaction will be discussed in greater detail in the subsequent paper by Cater and Taylor (1966).

SUMMARY

Tumour blood-flow changes, assessed by oxygen tension measurements and response to oxygen inhalation, often tended to mimic changes of systemic blood pressure except after injection of noradrenaline, adrenaline, or 5-hydroxytryptamine (5-HT). Noradrenaline, given by slow-perfusion, always raised the blood pressure but reduced tumour blood-flow. Adrenaline raised the blood pressure but the effects upon the tumour blood-flow were variable. The ganglion-stimulating agent dimethyl-phenyl-piperazine (DMPP) gave variable results as did hypertensin. Vasodilators studied included acetyl choline which increased tumour blood-flow, nicotinic acid gave variable results, while isoprenaline and amyl nitrite both decreased tumour blood-flow.

The inflammatory agents :—(1) 5-HT, stops tumour blood-flow ; (2) bradykinin, reduces blood-flow in tumour but increases it in tumour capsule ; (3) kallikrein (which produces bradykinin *in vivo*) has a similar effect.

We wish to thank, Mr. E. King of the Department of Radiotherapeutics for the supply of hepatoma-bearing rats, and Dr. I. A. Silver of the Department of Veterinary Anatomy for technical facilities.

### REFERENCES

Cater, D. B., Grigson, C. M. B. and Watkinson, D. A.—(1962) *Acta radiol.*, **58**, 401.
Cater, D. B., Petrie, A. and Watkinson, D. A.—(1965) *Acta radiol. Ther. Phys. Biol.*, **3**, 109.
Cater, D. B., Schoeniger, E. L. and Watkinson, D. A.—(1963) *Acta radiol. Ther. Phys. Biol.*, **1**, 233.
Cater, D. B. and Silver, I. A.—(1961) In ' Reference Electrodes ', edited by D. J. G. Ives and G. J. Janz. New York (Academic Press) p. 464.
Cater, D. B., Silver, I. A. and Wilson, G. M.—(1959) *Proc. R. Soc.* B., **151**, 256.
Cater, D. B. and Taylor, C. R.—(1966) *Br. J. Cancer*, **20**, 517.
Gullino, P. M. and Grantham, F. H.—(1962) *J. natn. Cancer Inst.*, **28**, 211.
Lewis, G. P.—(1964) *Ann. N.Y. Acad. Sci.*, **116**, 847.
Majno, G., Palade, G. E. and Schoefl, G. I.—(1961) *J. biophys. biochem. Cytol.*, **11**, 607.
Rocha e Silva, M.—(1964) *Ann. N.Y. Acad. Sci.*, **116**, 899.
Zweifach, B. W.—(1964) *Ann N.Y. Acad. Sci.*, **116**, 831.